# Linkages of Various Calcium Sources on Immune Performance, Diarrhea Rate, Intestinal Barrier, and Post-gut Microbial Structure and Function in Piglets

**DOI:** 10.3389/fnut.2022.921773

**Published:** 2022-06-17

**Authors:** Kaijun Wang, Anqi Yang, Xiaomin Peng, Feifei Lv, Ying Wang, Yao Cui, Yuhan Wang, Jianqun Zhou, Hongbin Si

**Affiliations:** ^1^State Key Laboratory for Conservation and Utilization of Subtropical Agro-Bioresources, College of Animal Science and Technology, Guangxi University, Nanning, China; ^2^Animal Nutritional Genome and Germplasm Innovation Research Center, College of Animal Science and Technology, Hunan Agricultural University, Changsha, China; ^3^Nanning Zeweier Feed Co., Ltd., Nanning, China

**Keywords:** piglet, calcium, bacterial community, feces, metabolites

## Abstract

The purpose of this experiment was to investigate the effects of different sources of calcium on immune performance, diarrhea rate, intestinal barrier, and post-intestinal flora structure and function in weaned piglets. A total of 1,000 weaned piglets were randomly assigned to five groups 10 replicate pens per treatment, 20 piglets per pen and fed calcium carbonate, calcium citrate, multiple calcium, and organic trace minerals of different concentrations of acidifier diets. The results of the study showed that the replacement of calcium carbonate with calcium citrate and multiple calcium had almost no significant effect on immune indexes (IL-1β, IL-6, IL-10, TNF-α) of piglets compared with the control group (*p* > 0.05). The five groups did not show a change in the diarrhea rate and diarrhea index (*p* > 0.05). The diet containing multiple calcium dramatically decreased the TP compared to the C and L diet (*p* < 0.05). No significant difference in HDL was noted in the five groups (*p* > 0.05). However, the concentration of LDL in blood in the multiple calcium group was significantly higher than that in groups L and D (*p* < 0.05). Moreover, the concentration of Glu in blood in the multiple calcium group was significantly higher than that in group C (*p* < 0.05). Compared with the control group, calcium citrate plus organic trace minerals diet markedly increased *UCG-005* abundance in the colon (*p* < 0.05). In addition, the relative abundance of *Prevotellaceae_NK3B31_group* had an upward trend in the colon of the M group compared to the D group (*p* = 0.070). Meanwhile, calcium citrate plus organic trace minerals diet markedly increased *Clostridium_sensu_stricto_1* abundance in the colon (*p* < 0.05). Metagenomic predictions by PICRUSt suggested that the colonic and fecal microbiota was mainly involved in carbohydrate metabolism, amino acid metabolism, energy metabolism, and metabolism of cofactors and vitamins.

## Introduction

The immune system of newborn piglets is not mature enough, and it mainly depends on the protection of maternal antibodies in pig milk before weaning (1). Antibiotics are often added to pigs’ feed to reduce the occurrence of diseases, such as diarrhea, and to improve growth performance. However, there are growing concerns about the risk of antibiotic-resistant bacteria and the residual effects of antibiotics in meat products. Subsequently, the use of antibiotics as growth promoters for sub-therapeutics has been banned in Europe since 2006 (Council Regulation EC 70/524/EEC). Therefore, there are growing interests in finding suitable alternatives to antibiotics. The weaning transition in piglets is a stressful process associated with decreased feed intake, poor performance, and increased susceptibility to infection, including post-weaning diarrhea (2, 3). After weaning, due to changes in the feeding environment and diet, pathogenic microorganisms, such as bacteria, viruses, and fungi, can damage immune organs and cells, interfere with antigen presentation, and inhibit or block the production of antibodies, resulting in a decline in the ability of piglets to resist disease and cause immunosuppression (4–6). An earlier study showed that in-feed antibiotic supplementation in weaned pigs reduced the microbiota diversity of colonic digesta (7). Minerals and vitamins are trace substances essential for the normal physiological function of animals, but their effects on intestinal flora are not well understood. Calcium is an essential element in biology; participates in the construction of tissues, such as bones, muscles, nerves, and body fluids; and is involved in important physiological processes, such as nerve transmission, muscle contraction, and hormone secretion (8). As a second messenger, calcium ions are involved in the signal transmission between immune cells, such as B lymphocytes, T lymphocytes, macrophages, and mast cells (9). Phosphorus is also an important element in the process of metabolism and signal transduction and is involved in bone metabolism and immune regulation together with calcium (10). Dietary supplementation with P and Ca has been suggested as a potential strategy to modulate the gastrointestinal tract microbiota in pigs based on studies with rats (11), where a decreased abundance of pathogens and promotion of lactobacilli in the small intestine in Ca- and P-rich diets have been observed (12). Studies have shown that calcium supplementation can significantly relieve colon inflammation and immune response caused by high-fat diet (13); increasing the concentration of intracellular calcium ions can significantly increase the function of cytotoxic T lymphocytes and natural killer cells (14). Therefore, according to previous research results, it is speculated that appropriate calcium supplementation can help improve the immune function of the body and alleviate the impact of immunosuppression on the growth and development of piglets, but there is a lack of relevant research at present.

Animal bodies, especially the digestive, urinary, respiratory, and reproductive tracts, are permanently colonized by millions of phylogenetically and metabolically diverse microscopic life forms (microbiota) (15). These microbes are critical for many key biological processes in their hosts, such as immune processes and digestive pathways, that are necessary for the maintenance of health and wellbeing (16, 17). The number of genes present in the microbial genome of the digestive tract of monogastric animals is about 100 times that of the body itself (18). Due to the large number and complex functions of microorganisms in the digestive tract, they are regarded as a “mobile organ” in animals, mediating or affecting physiological processes, such as host nutrient metabolism and immunity (19). This close microbiota–host relationship is the result of the synchronized evolution of these two life forms over millions of years, a complex process known as co-evolution (20, 21), in which microbes evolve according to host characteristics, such as diet and gut type, to establish communities and are closely linked to the immune system (22, 23). Mammalian gut microbial community is a heterozygous ecosystem and composed of thousands of microbial species (24). Gut microbiota influences many important host physiological functions, such as modulation of food intake, metabolism, immune system activation, epithelial cell proliferation, and resistance to infection (25). The microbiota structure in the cecum is well studied, while the colon and feces are poorly understood. Cecum is the place with the most abundant microorganism species and content in single-stomach animals. The number of microbes per gram of intestinal content in pigs is 10^12^∼10^13^ CFUs and composed of 400∼500 species of microorganisms, which mainly consist of *Bacteroides* (8.5∼27.7%), clostridium X and IV of Firmicutes (10.8∼29.0%), and clostridium IV (25.2%) as the predominant flora (26–28). The stable intestinal microbial flora can form a bacterial membrane barrier on the surface of intestinal epithelial cells to help the host resist harmful foreign bacteria or inhibit the invasion and reproduction of intestinal pathogenic bacteria by competing for nutrients (29). At the same time, the stable microbial flora in the intestine participates in the host’s nutrient metabolism through fermentation, degradation of polysaccharides, and synthesis of vitamins (30, 31). The main objective of the current study was to evaluate the effect of different combinations of post-weaning calcium and acidifier supplementation on immune performance, diarrhea rate, intestinal barrier, and post-gut microbial structure and function of weaned piglets.

## Experimental Design

The studies were approved by the Laboratory Animal Welfare and Animal Experimental Ethical Inspection Committee at the Guangxi University (Nanning, China).

### Animals, Diets, and Management

Briefly, a total of 1,000 piglets (Yorkshire × Landrace), weaned at the age of 21 days with a mean body weight (BW) of 6.09 ± 0.26 kg, were randomly assigned to one of five dietary treatments with 20 replicate pens (50 piglets per replicate pen) for 42 days. Water and feed were provided *ad libitum*. Diarrhea in piglets in each group was recorded every day.

The compositions of the basal diets are given in Table 1. Experiment diet (Table 2) was formulated to provide different dietary concentrations of calcium from calcium carbonate, calcium citrate, or multiple calcium. Calcium carbonate (Ca ≥ 36.65%), calcium citrate (Ca ≥ 23.4%), and multiple calcium (Ca ≥ 24%) used in this experiment were all provided by Nanning Zeweier Feed Co., Ltd. The formula of diet should meet or exceed the nutritional needs of weaned piglets (32). L = C (control) plus 5/1,000 calcium citrate replace calcium carbonate; D = L plus 1/1,000 organic trace minerals; M = D minus half of the acidifier; P = C (control) plus 5/1,000 multiple calcium replace calcium carbonate. The diarrhea index score of feces is given in Supplementary Table 1. Piglets should be housed on a 12 h light/12 h dark cycle with free access to water, and the barn temperature was maintained at 30°C.

**TABLE 1:** Ingredients and composition of the basal diet for C (control).

Ingredients,%	0–14 days	14–42 days
Corn	61.43	65.02
Soybean meal	6.41	8.21
Extruded soybean	6.12	4.25
Whey powder	12.00	10.00
Fish meal	5.00	4.00
SDPP	5.00	3.00
Limestone	0.90	0.80
Dicalcium phosphate	0.40	0.54
Salt	0.35	0.30
L-lysine HCl (98%)	0.33	0.45
DL-Methionine	0.11	0.07
L-Threonine	0.11	0.13
L-Tryptophan	0.01	0.03
Soy oil	1.28	2.76
ZnO	0.20	0.04
Vitamin and mineral premix^a^	0.35	0.40
**Nutrient composition (%)**		
DE (Kcal/kg)	3455	3452
CP	19.41	19.06
Calcium	0.85	0.84
Phosphorus	0.32	0.32

*SDPP, spray-dried plasma protein; ^a^vitamin–mineral premix supplied per kg of feed: 100 mg of Fe (FeSO4), 100 mg of Zn (ZnSO4), 30 mg of Mn (MnSO4), 25 mg of Cu (CuSO4), 0.5 mg of I (KIO3), 0.3 mg of Co (CoSO4), 0.3 mg of Se (Na2SeO3), and 0.5 mg of ethoxyquin, 10,500 IU of vitamin A, 200 IU of vitamin D3, 60 IU of vitamin E, 2.0 mg of vitamin K3, 0.03 mg of vitamin B12, 12 mg of riboflavin, 30 mg of niacin, 25 mg of D-pantothenic acid, 0.18 mg of biotin, 1.5 mg of folic acid, 3.0 mg of thiamine, 2.25 mg of pyridoxine, and 500 mg of choline chloride.*

**TABLE 2:** Experimental diets for one thousand weaned piglets.

Items	Groups				
	
	C (*n* = 200)	L (*n* = 200)	D (*n* = 200)	M (*n* = 200)	P (*n* = 200)
Calcium carbonate (kg)	normal	–	–	–	–
Calcium citrate (kg)	–	5/1,000	5/1,000	5/1,000	–
Acidifier (kg)	1/1,000	1/1,000	1/1,000	0.5/1,000	1/1,000
Organic trace minerals (kg)	–	–	1/1,000	1/1,000	1/1,000
Multiple calcium (kg)	–	–	–	–	5/1,000

*C, Basal diet (Calcium carbonate + 1/1,000 g/kg of acidifier);*

*L, 5/1,000 Calcium citrate diet + 1/1,000 g/kg of acidifier;*

*D, 5/1,000 Calcium citrate diet + 1/1,000 g/kg of acidifier + 1/1,000 g/kg organic trace minerals;*

*M, 5/1,000 Calcium citrate diet + 0.5/1,000 g/kg of acidifier + 1/1,000 g/kg organic trace minerals;*

*P, 5/1,000 Multiple calcium diet + 1/1,000 g/kg of acidifier + 1/1,000 g/kg organic trace minerals.*

### Sampling and Collection

At the end of the experiment on day 42, two blood samples were collected using heparin tubes from the front cavity vein of eight weaned piglets in five group separately. Collected plasma samples were centrifuged at 1,000 g for 15 min at 4°C and stored at −20°C for further analysis. Four weaned piglets were euthanized in each group, and intestinal and fecal samples were subsequently collected. The colonic chyme and fecal sample were gathered and stored separately at −80°C for DNA extraction. The segments of the duodenum, jejunum, and ileum were taken for observation of intestinal tissue morphology. The samples of the duodenal, jejunal, and ileal segments from weaned piglets were fixed in formalin, and the tissues were dehydrated and embedded following standard procedures; specimens in paraffin block were cut into 5 μm sections and stained with hematoxylin and eosin. The representative photographs of the duodenal, jejunal, and ileal morphology were collected using an optical microscope with a Pannoramic Scannera computer-aided morphometry system. In the present study, we used a pre-defined method reported by Wang et al. (33) to define the lesion. In each section, the villus height (VH) and crypt depth (CD) were measured using a light microscope with a computer-assisted morphometric system. The VH was defined as the distance from the villus tip to the crypt mouth, and the CD from the crypt mouth to the base.

### Metabolite Measure in the Plasma

Two piglets were selected in each repeat of five groups (40 piglets in all), and a total of 40 plasma samples were used for analysis. The plasma biochemical components, including total protein (TP), albumin (ALB), low-density lipoprotein (LDL), high-density lipoprotein (HDL), triglyceride (TG), glucose (Glu), blood urea nitrogen (BUN), interleukin 1β (IL-1β), interleukin 6 (IL-6), interleukin 10 (IL-10), and tumor necrosis factor α (TNF-α), were detected using the enzyme-linked immunosorbent assay (ELISA) kits (Jiangsu Meimian industrial Co., Ltd, Yancheng, China) following the manufacturer’s instructions.

### Colon and Feces Content of Microflora by 16S rRNA Sequencing

Totally, 20 piglets were selected in each repeat of five groups for colon microbiota analysis, and 40 piglets were selected in each repeat of five groups for feces microbiota analysis. Microbial DNA was extracted from approximately 0.25 g of each intestinal sample and fecal sample using a QIAamp DNA Stool Mini Kit, following the manufacturer’s instructions (34). DNA isolation was performed by 2% agarose gel electrophoresis. The bacterial universal V3–V4 regions of the 16S rRNA gene were amplified according to PCR-barcoded primers 338F (5′-ACTCCTACGGGAGGCAGCAG-3′) and the reverse primer 806R (5′-GGACTACHVGGGTWTCTAAT-3′). The specific sequencing method was used as previously reported (35). The thermal cycle procedure is as follows: initial denaturation step, 95°C, 3 min; denaturation, 27 cycles, 95°C, 30 s; annealing, 55°C, 30 s; elongation, 72°C, 45 s; and final extension, 72°C, 10 min. Briefly, paired-end sequenced on an Illumina MiSeq platform (PE300) platform (Illumina, United States) at the Majorbio Bio-Pham Technology (Shanghai, China). The 16S rRNA amplified sequences have been deposited in the National Center for Biotechnology Information (NCBI) Sequence Read Archive (SRA)^1^ under accession number PRJNA815982.

### Microbiome Analysis

Quality filters were applied to trim the original sequences according to the criteria: (I) reads with an average quality score <20 over a 10-bp sliding window were removed, and truncated reads smaller than 150 bp were discarded and (II) truncated reads containing homopolymers longer than eight nucleotides in length, more than 0 base in barcode matches, or more than two different bases in primers were removed from the dataset. Checking and removing possible chimeras by USEARCH using the chimera layer “gold” database described by Edgar et al. (36). Clustering of OTUs with a similarity cutoff of 97% using USEARCH (37), and abundance-defining representative sequences for each OTU were identified using PyNAST (38) and the SILVA bacterial database (39). The rarefaction analysis was performed by Mothur v.1.39.5 (40) to reveal diversity indexes, including the Chao index and Shannon index. PCoA was performed using Canoco 4.5. Venn diagrams were implemented by Venn diagrams, and community diagrams were generated by R tools from the data in the files “tax. phylum.xls, tax.family.xls, and tax. genus.xls.”

### Predictive Functional Profiling of Microbial Communities

PICRUSt has been used as a bioinformatics tool to predict the functional potential of metagenomes using 16S rRNA genetic data (41). Subsequently, by referencing the KEGG database, the OTU table was imported into PICRUSt for functional gene prediction. PICRUSt utilizes 16S copy number prediction to normalize the OTU table so that OTU abundance more accurately reflects the true abundance of the underlying organism. We then looked for the precomputed genome content of each OTU, multiplied the normalized OTU abundance by each KEGG abundance in the genome, and summed these KEGG abundance for each sample to predict the metagenome. This prediction calculates the KEGG abundance for each metagenomic sample in the OTU table. For those optional organism-specific predictions, each organism abundance per KEGG is kept and annotated. We focused our exploration of metagenomes at levels 2 and 3. These pathways related to organismal systems, human disease, and drug development were filtered out because they do not reflect microbial function.

### Statistical Analyses

Statistical analyses between the means of each group were analyzed by using one-way analysis of variance (ANOVA), followed by multiple comparisons using a *post hoc* test of S-N-K through SPSS 22.0. Statistical significance was set at *p* < 0.05.

## Results

### Intestinal Histomorphological Analysis

**FIGURE 1:**
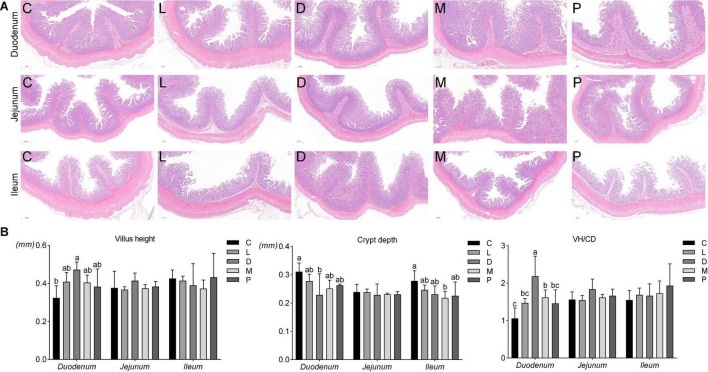
Effects of different diet on intestinal morphology in weaned piglets. **(A)** Effects of different diets on epithelial morphology of the duodenum, jejunum, and ileum. **(B)** Effects of different diets on the villus length and crypt depth of the duodenum, jejunum, and ileum. C, Basal diet (Calcium carbonate + 1/1,000 g/kg of acidifier); L, 5/1,000 Calcium citrate diet + 1/1,000 g/kg of acidifier; D, 5/1,000 Calcium citrate diet + 1/1,000 g/kg of acidifier + 1/1,000 g/kg organic trace minerals; M, 5/1,000 Calcium citrate diet + 0.5/1,000 g/kg of acidifier + 1/1,000 g/kg organic trace minerals; P, 5/1,000 Multiple calcium diet + 1/1,000 g/kg of acidifier + 1/1,000 g/kg organic trace minerals.

Cross-sections of intestinal tissue samples were stained with hematoxylin and eosin and observed under a light microscope. The duodenal, jejunal, and ileal tissue morphologies under five different dietary treatments are shown in Figure 1A. The addition of calcium carbonate to the diet of weaned piglets resulted in a lower villus height in the duodenum than other calcium sources, and the crypt depth of the piglets in the diet supplemented with calcium carbonate was higher. From Figure 1B, it can be concluded that dietary replacement of different calcium sources had no significant effect on the jejunal villus height and crypt depth, and the ratio of villus height to crypt depth in weaned piglets (*p* > 0.05). Compared with the control group, the D group significantly increased the villus height and significantly decreased the crypt depth (*p* < 0.05). At the same time, the M group significantly reduced the crypt depth of the ileum compared with the control group (*p* < 0.05). Finally, the ratio of villus height to crypt depth of the duodenum in group D was significantly higher than that of the other four groups (*p* < 0.05), and the ratio of villus height to crypt depth in group M was also significantly higher than that of the control group (*p* < 0.05).

### Diarrhea Rate and Diarrhea Index

As shown in Table 3, compared with basal diet, diet of D varied with the decreasing diarrhea rate of weaned piglets on days 1–14 (*p* = 0.083). However, there was no significant difference between different treatment groups during the whole experimental period (*p* > 0.10). Correspondingly, there was no significant difference in the diarrhea index under the treatment of five diets during the whole experimental period (*p* > 0.10).

**TABLE 3:** Effects of different calcium sources on diarrhea rate and diarrhea index of weaned piglets.

Items	Groups				
	
	C (*n* = 200)	L (*n* = 200)	D (*n* = 200)	M (*n* = 200)	P (*n* = 200)
**Diarrhea rate**					
Day 1–14	1.78 ± 0.87	1.38 ± 1.02	0.93 ± 0.29	1.28 ± 0.42	1.03 ± 0.19
Day 15–28	1.65 ± 1.30	1.58 ± 1.47	1.18 ± 0.43	0.88 ± 0.73	1.08 ± 0.34
Day 29–42	5.03 ± 1.23	4.73 ± 1.18	4.93 ± 1.88	5.33 ± 1.62	5.15 ± 0.77
**Diarrhea index**					
Day 1–14	0.68 ± 0.28	0.52 ± 0.32	0.39 ± 0.14	0.39 ± 0.24	0.39 ± 0.14
Day 15–28	0.55 ± 0.43	0.55 ± 0.49	0.36 ± 0.12	0.30 ± 0.26	0.32 ± 0.12
Day 29–42	1.11 ± 0.18	0.91 ± 0.07	0.96 ± 0.33	1.04 ± 0.19	1.04 ± 0.04

*Diarrhea rate (%) = the number of diarrhea pigs × diarrhea day/(the total number of pigs × experiment days); in the same row, values with no letter or the same letter superscripts mean no significant difference (p > 0.05), while values with different small letter superscripts mean significant difference (p < 0.05).*

### Biochemical Parameters in the Plasma

**FIGURE 2:**
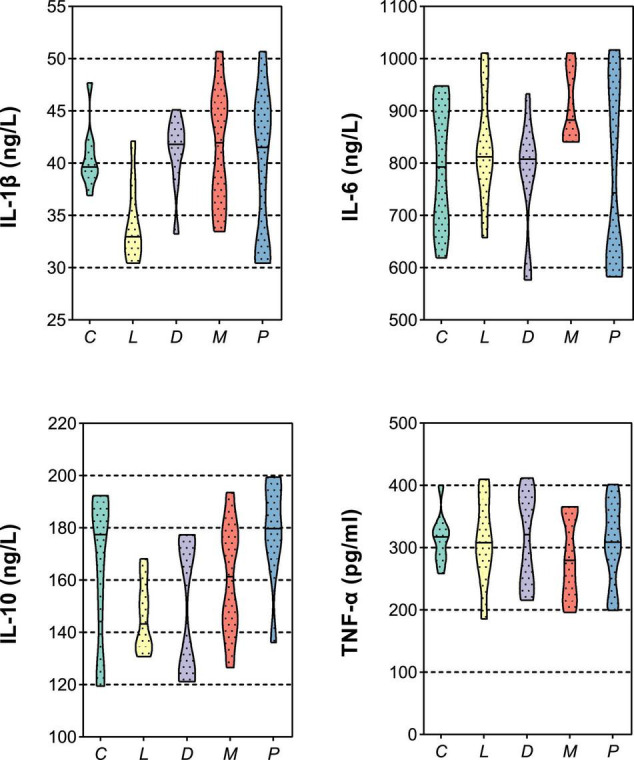
Effects of different diets on the blood immune index in weaned piglets. IL-1β, interleukin 1β; IL-6, interleukin 6; IL-10, interleukin 10; TNF-α, tumor necrosis factor α.

Variation in the biochemical index in the plasma is given in Table 4. Diet of multiple calcium dramatically decreased the TP compared to the C and L diet (*p* < 0.05). There were no significant effects of including different sources of calcium in diets with normal or halved acidifier fed piglets on ALB in 6 weeks (*p* > 0.05). Meanwhile, no significant difference in HDL was noted among the five groups (*p* > 0.05). However, the concentration of LDL in blood in the multiple calcium group was significantly higher than that in groups L and D (*p* < 0.05). Moreover, the concentration of Glu in blood in the multiple calcium group was significantly higher than that in group C (*p* < 0.05). It was proved that piglet feed calcium carbonate, calcium citrate, multiple calcium, or different amount of acidifier had no influence on the content of TG and BUN (*p* > 0.05). A similar case occurs in immune function of blood (Figure 2); there was no significant difference in immune indexes (IL-1β, IL-6, IL-10, and TNF-α) among different treatments after weaning 6 weeks for piglets (*p* > 0.05).

**TABLE 4:** Effects of different calcium sources on plasma biochemical indexes of weaned piglets.

Items	Groups				
	
	C (*n* = 8)	L (*n* = 8)	D (*n* = 8)	M (*n* = 8)	P (*n* = 8)
TP (μg/L)	0.986 ± 0.175^a^	1.003 ± 0.156^a^	0.908 ± 0.212^ab^	0.940 ± 0.211^ab^	0.779 ± 0.120^b^
ALB (μg/ml)	107.0 ± 16.6	105.1 ± 17.8	104.0 ± 10.5	103.9 ± 15.5	116.9 ± 18.9
LDL (μmol/L)	244.9 ± 38.0^ab^	227.1 ± 20.1^b^	227.6 ± 44.1^b^	246.9 ± 18.6^ab^	264.6 ± 23.9^a^
HDL (μmol/L)	56.19 ± 6.96	58.21 ± 3.99	57.92 ± 7.15	61.88 ± 4.96	56.29 ± 5.78
TG (μmol/L)	421.4 ± 92.6	388.0 ± 46.6	390.1 ± 90.3	451.0 ± 77.8	424.9 ± 81.4
Glu (μmol/L)	109.3 ± 14.2^b^	122.6 ± 18.1^ab^	123.8 ± 19.0^ab^	118.5 ± 19.9^ab^	130.8 ± 16.5^a^
BUN (mol/L)	1.32 ± 0.28	1.44 ± 0.29	1.42 ± 0.28	1.40 ± 0.29	1.33 ± 0.23

*TP, total protein; ALB, albumin; LDL, low-density lipoprotein; HDL, high-density lipoprotein; TG, triglyceride; Glu, glucose; BUN, blood urea nitrogen. In the same row, values with no letter or the same letter superscripts mean no significant difference (p > 0.05), while values with different small letter superscripts mean significant difference (p < 0.05).*

### Diet and Gastrointestinal pH

Effects of different calcium sources on diet and gastrointestinal pH of weaned piglets are shown in Table 5. Diet pH of weaned piglets did not differ among the five dietary treatments in 6 weeks (*p* > 0.05). After the weaned piglets were slaughtered after feeding for 6 weeks, there was no significant difference in the pH values of the stomach, small intestine, and large intestine.

**TABLE 5:** Effects of different calcium sources on diet and gastrointestinal pH of weaned piglets.

Items	Groups				
	
	C	L	D	M	P
**Diet**					
2 weeks	5.44	5.71	5.72	5.74	6.01
4 weeks	5.78	5.86	5.87	5.88	6.21
6 weeks	5.66	5.61	5.64	5.65	6.08
**Gastrointestinal tract**					
Stomach	6.48 ± 0.34	6.21 ± 0.41	6.22 ± 0.48	6.04 ± 0.89	5.31 ± 1.31
Duodenum	6.41 ± 0.31	6.38 ± 0.19	6.40 ± 0.35	6.38 ± 0.15	6.88 ± 0.43
Jejunum	6.41 ± 0.21	6.39 ± 0.36	6.59 ± 0.11	6.73 ± 0.39	6.65 ± 0.16
Ileum	6.83 ± 0.36	6.70 ± 0.27	6.75 ± 0.21	6.78 ± 0.19	6.90 ± 0.22
Colon	6.56 ± 0.11	6.67 ± 0.33	6.42 ± 0.33	6.66 ± 0.09	6.34 ± 0.08

*In the same row, values with no letter or the same letter superscripts mean no significant difference (p > 0.05), while values with different small letter superscripts mean significant difference (p < 0.05).*

### Colonic and Fecal Bacterial Diversity and Similarity

**FIGURE 3:**
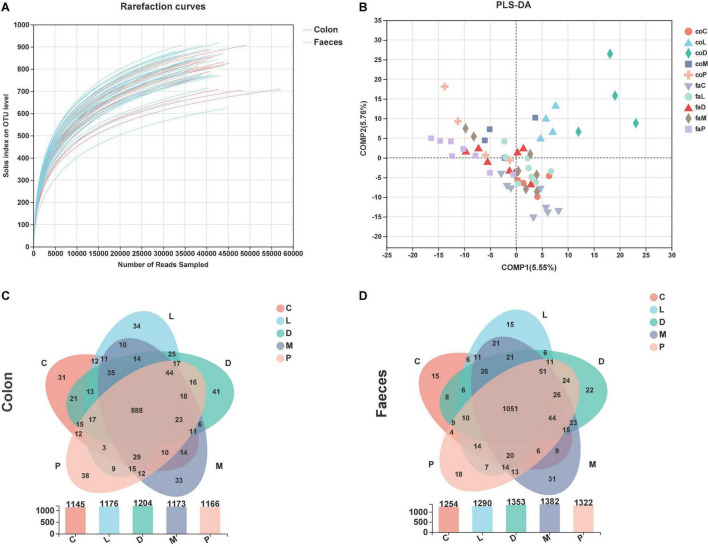
Bacteria richness and OTU composition analysis. **(A)** Rarefaction curves of observed bacterial sequences in the colonic contents and feces of piglets. **(B)** Partial least squares-discriminant analysis (PLS-DA) of colonic digestive and fecal bacterial community. **(C)** Venn diagram of the OTUs in the colon by different treatments. **(D)** Venn diagram of the OTUs in the feces by different treatments.

The rarefaction curves reached a plateau, suggesting that the selected sequences were reasonable and fully measured most of the bacterial diversity (Figure 3A). The OTU community comparisons by partial least squares-discriminant analysis (PLS-DA) of colonic digestive and fecal bacterial communities showed that 60 samples from colon and feces were well separated in some groups (Figure 3B). From the colonic samples, we could see only the coM and coP groups did not show a good separation, and groups stayed away in the other three groups. The faM and faP groups showed a separation, and the other three sets of samples were mixed together (Figure 3B).

As shown in Figure 3C, the overall OTU numbers classified at the distance level of 0.03 (97% similarity) were 1,477 detected in the colonic samples, most abundance with 1,204 OTUs was observed in the D group, and the control group had the least 1,145 OTUs, and 888 were shared in five group. The fecal OTU numbers were more than colon OUTs and had 1,557 OTUs (Figure 3D). Similarly, the control group of the feces had the least OTU number, and 1,051 OTUs were shared in the feces by five diet treatments.

**FIGURE 4:**
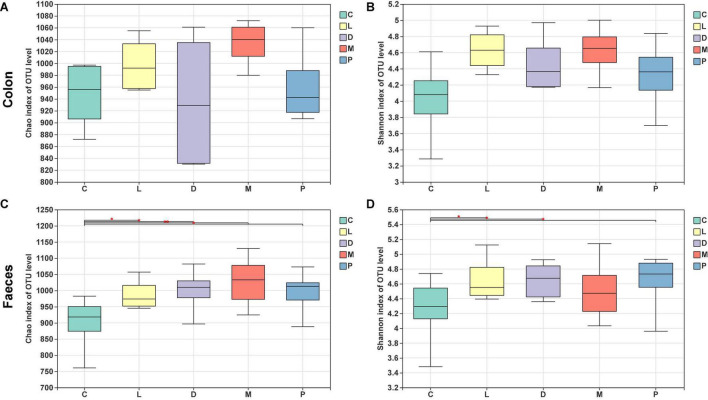
Alpha-diversity of colonic and fecal bacterial communities of piglets. **(A)** Bacterial richness in the colon estimated by the Chao 1 value. **(B)** Bacterial diversity in the colon estimated by the Shannon index. **(C)** Bacterial richness in the feces estimated by the Chao 1 value. **(D)** Bacterial diversity in the feces estimated by the Shannon index.

The bacterial community of the M group had the higher Chao1 estimator and Shannon diversity index than the other four groups, and the D group showed the lowest Chao1 estimator in the colon (Figures 4A,B). However, there was no significant difference in the two indicators among the five groups (*p* > 0.05). In fecal samples, the C group showed the lowest Chao1 estimator than the other four diet treatment groups (Figure 4C), and there was a significant difference for the Chao1 estimator in the control group compared to the other four groups (*p* < 0.05). Meanwhile, as shown in Figure 4D, groups L, D, and P had markedly improved Shannon index than the control group (*p* < 0.05), and there was no significant change in the Shannon index in groups C and M (*p* > 0.05).

### Taxonomic Composition by Illumina MiSeq Sequencing Analysis

**FIGURE 5:**
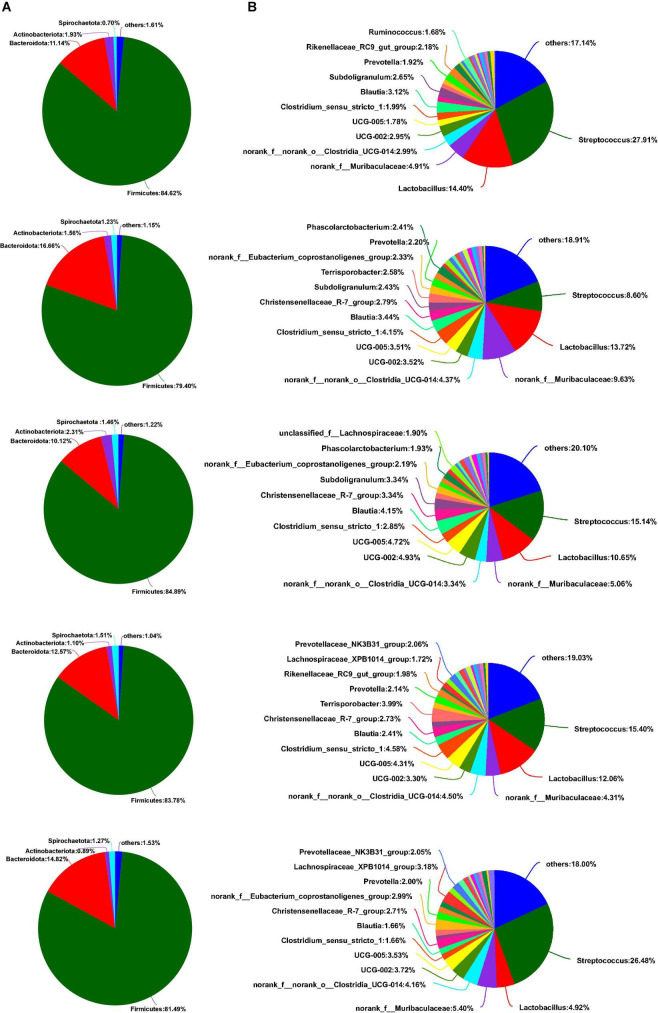
Effects of different diets on the colonic bacterial community structure in weaned piglets. **(A)** Distribution of colonic bacteria at the phylum level in weaned piglets. **(B)** Distribution of colonic bacteria at the genus level in weaned piglets.

**FIGURE 6:**
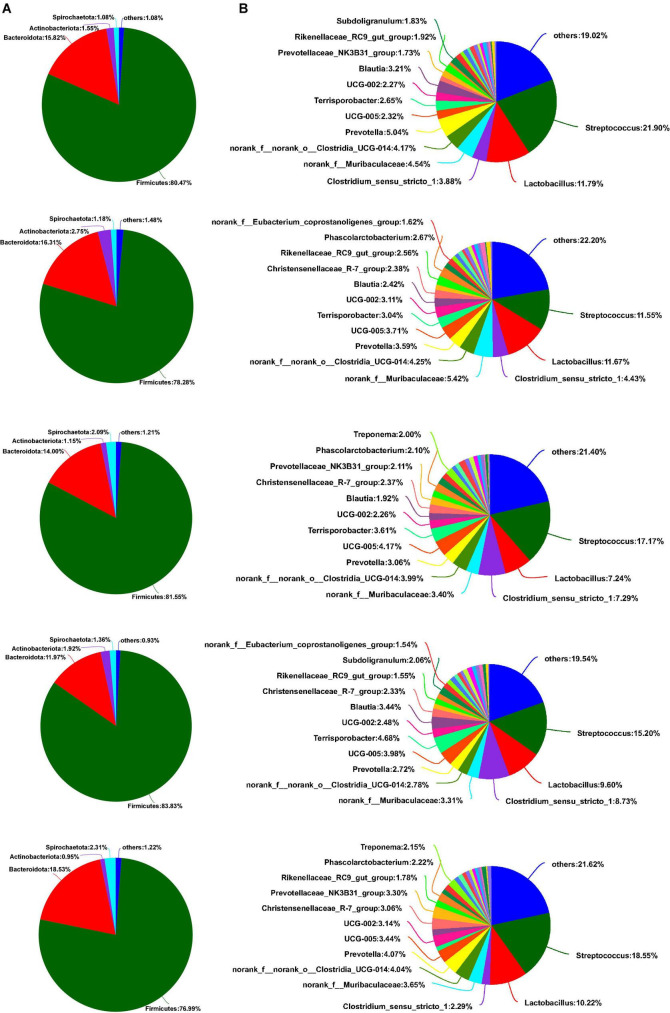
Effects of different diets on the fecal bacterial community structure in weaned piglets. **(A)** Distribution of fecal bacteria at phylum level in weaned piglets. **(B)** Distribution of fecal bacteria at the genus level in weaned piglets.

As shown in Figure 5A, Firmicutes, Bacteroidota, Actinobacteriota, and Spirochaetota were dominant phyla in the colon of weaned piglets, accounting for more than 98% of the colonic total bacterial community. Although some bacterial communities of phyla proportion varied with the different sources of calcium added to the diet, there was no significant difference between the main bacterial communities at the phyla level in the colon (*p* > 0.05). Downward to genus levels, *Streptococcus*, *Lactobacillus*, *norank_f_Muribaculaceae, norank_f_norank_o_Clostridia_UCG-014*, and *UCG-002* were the predominant genera in the colon in the five groups (Figure 5B). Compared to the colon, the most abundant bacterial community in the feces at the phylum level was Firmicutes, followed by Bacteroidota, Actinobacteriota, and Spirochaetota, from most to least (Figure 6A). Similarly, no relative abundance of bacteria at the phylum level was significantly changed in the feces of the five groups (*p* > 0.05). Downward to the genus level, *Streptococcus*, *Lactobacillus*, *Clostridium_sensu_stricto_1*, *norank_f_Muribaculaceae*, and *norank_f_norank_o_Clostridia_UCG-014* were the predominant genera in feces in the five groups (Figure 6B).

**FIGURE 7:**
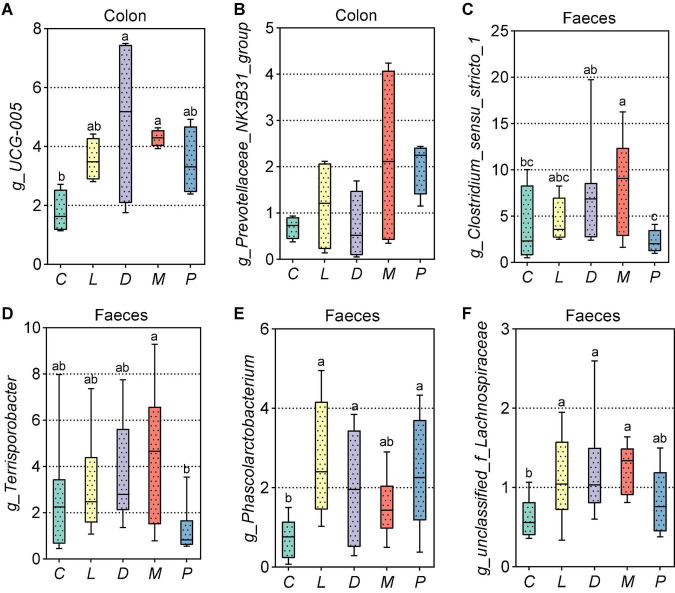
Effects of different diets on intestinal and fecal different bacteria in weaned piglets. **(A,B)** Top 30 different bacteria at the genus level of the colon in weaned piglets. **(C–F)** Distribution of top 30 different fecal bacteria at the genus level in weaned piglets. C, Basal diet (Calcium carbonate + 1/1,000 g/kg of acidifier); L, 5/1,000 Calcium citrate diet + 1/1,000 g/kg of acidifier; D, 5/1,000 Calcium citrate diet + 1/1,000 g/kg of acidifier + 1/1,000 g/kg organic trace minerals; M, 5/1,000 Calcium citrate diet + 0.5/1,000 g/kg of acidifier + 1/1,000 g/kg organic trace minerals; P, 5/1,000 Multiple calcium diet + 1/1,000 g/kg of acidifier + 1/1,000 g/kg organic trace minerals.

As shown in Figure 7A, compared with the results of the control group, calcium citrate plus organic trace mineral diet markedly increased *UCG-005* abundance in the colon (*p* < 0.05). In addition, the relative abundance of *Prevotellaceae_NK3B31_group* had an upward trend in the colon of the M group compared to the D group (*p* = 0.070) (Figure 7B). As can be seen from Figure 7C, replacing calcium citrate with multiple calcium significantly reduced the abundance of *Clostridium_sensu_stricto_1* (*p* < 0.05), and calcium citrate plus organic trace minerals diet markedly increased *Clostridium_sensu_stricto_1* abundance in the colon (*p* < 0.05). The addition of multiple calcium, instead of calcium citrate and the acidifier with half reduction, could significantly reduce the related abundance of *Terrisporobacter* (*p* < 0.05) (Figure 7D). From Figures 7E,F, we could see that the addition of five thousandths of calcium citrate plus one thousandth of acidifier in the diet significantly increased the related abundance of *Phascolarctobacterium* and *unclassified_f_Lachnospiraceae* compared with the calcium carbonate diet (*p* < 0.05).

### Biofunction Prediction of Intestinal and Fecal Microbial Flora

**FIGURE 8:**
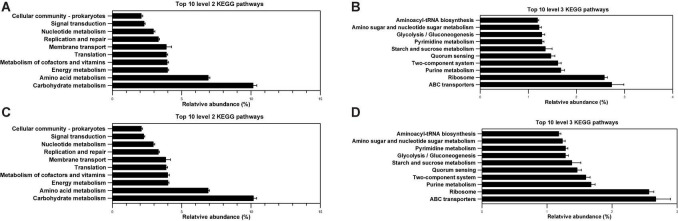
Effects of different diets on predicted metagenomic functions of the KEGG pathway in weaned piglets. **(A,B)** Top 10 predicted metagenomic functions at level 2 **(A)** and level 3 **(B)** of the KEGG pathways in colon. **(C,D)** Top 10 predicted metagenomic functions at level 2 **(C)** and level 3 **(D)** of the KEGG pathways in feces.

In the study, PICRUSt was used to analyze the microbiota function of the ileum and cecum. The 16S rRNA sequencing results combined with genomic databases could be used to predict macrogenomic information (41). The predictable results can be enriched at two different levels of the KEGG pathways in the colon, where 2 and 3 level impressions are used for histograms (Figure 8). As shown in Figure 8A, within the top 10 KEGG pathways, membrane transport and signal transduction pathway were associated with environmental information processing. Five other pathways, including the metabolism of carbohydrates, amino acids, energy, cofactors and vitamins, and nucleotides, were associated with nutrient metabolism. Translation, replication, and repair were associated with genetic information processing. The cellular community prokaryotes were associated with cellular processes. In total, 318 individual pathways were predicted, and the top 10 most abundant pathways included three pathways related to carbohydrate metabolism (as shown in Figure 8B), including amino sugar and nucleotide sugar metabolism (ko00520), glycolysis/gluconeogenesis (ko00010), and starch and sucrose metabolism (ko00500). The highest abundance was ABC transporters (ko02010), and it belonged to membrane transport. In addition, both of aminoacyl-tRNA biosynthesis (ko00970) and ribosome (ko03010) belonged to translation, and 342 individual pathways were totally predicted in the feces. As shown in Figure 8C, the prediction results on level 2 of the KEGG pathway in the feces was similar to those in the colon, only the proportion is slightly different. The abundant KEGG pathway of glycolysis/gluconeogenesis (ko00010) in the feces was higher than pyrimidine metabolism (ko00240), which was contrary to the result in the colon (Figure 8D). Lastly, there was no significant difference in the 2 and 3 levels of the top 10 KEGG pathways in the colon and feces (*p* > 0.05).

## Discussion

Premature weaning of piglets can cause stress, most commonly diarrhea, resulting in huge economic losses in pig production. In recent years, studies have found that regardless of causes of piglet weaning diarrhea, the diversity of intestinal flora will decrease and the structure will change. A stable gut flora not only protects against diarrhea-causing pathogens, such as ETEC, *Clostridium difficile*, *Salmonella typhimurium*, and other diarrhea-causing pathogens (42), but also plays an important role in regulating animal health, such as immune response, intestinal barrier, intestinal muscle reflex, and endocrine (43).

The small intestine is an important place for the body to absorb nutrients. Villus height and crypt depth are important indicators to measure the digestion and absorption function of the small intestine. The depth of the crypts reflects the rate of cell formation, while shallower crypts indicate an increased rate of cell maturation and enhanced secretory function. The height of villi and the depth of crypts can comprehensively reflect the functional status of the small intestine (44). In the present study, after weaned piglets were fed different calcium sources and different ratios of acidifier for 6 weeks, the structure of the small intestinal epithelium of piglets fed calcium citrate and organic trace elements was significantly changed. Different calcium sources did not significantly alter the villus height and crypt depth of the jejunum. The use of calcium citrate plus organic trace elements and half the acidifier only caused a decrease in the ileal crypt depth compared to the control group but had no effect on the villus height. The duodenum of the four experimental groups had higher villus height and lower crypt depth than the control group. Overall, the results of the study showed that the small intestine of piglets fed a diet with calcium citrate and organic trace elements had a better effect. Therefore, these findings suggest that calcium citrate diets are superior to calcium carbonate diets for piglets.

Gastrointestinal acidity is one of the important indicators to judge the digestive environment of the animal gastrointestinal tract, and it is also an important factor to ensure the normal physiological function of gastrointestinal microorganisms. Beneficial bacteria, such as *Lactobacillus*, are suitable for growth in an acidic environment, while pathogenic microorganisms, such as ETEC and *Salmonella*, can survive in a neutral alkaline (pH 6.0–8.0) environment. After early weaning of piglets, due to the decrease in the concentration of lactic acid bacteria in the digestive tract, the pH of the gastrointestinal tract will increase compared with that before weaning, which is likely to cause a decrease in intestinal acidity and the activity of digestive enzymes in the small intestine (45). Coupled with the stress caused by changes in the diet and environment, this is conducive to the proliferation of pathogenic bacteria, resulting in nutritional or pathogenic diarrhea in piglets (46). Li et al. showed that reducing the level of *E. coli* in the gut microbiota can reduce the incidence of diarrhea in piglets (47). Organic acids have long been used to support piglet’s growth, especially at weaning, and have recently become the preferred alternative to growth promoters that increase piglet’s productivity (48). Organic acids in feed have been reported to be effective growth promoters in pigs throughout the production cycle, although due to the type and dosage of organic acids used, timing of supplementation, type of diet, buffering capacity, hygiene and welfare standards, health status, animal age, and other factors, the response is quite different (49). This experiment proved that the diarrhea rate and diarrhea index of piglets did not change significantly with the addition of different calcium sources and the use of acidifiers.

Blood physiological and biochemical indicators can not only reflect the health of the animal body and the strength of immune function but also reflect the biological characteristics of different animals (50). The level of serum TP content will reflect the strength of animal immunity, which will affect the absorption and utilization of animal nutrients. In this study, the addition of multiple calcium decreased the TP content of piglets compared with the other four treatments, indicating that multiple calcium may be detrimental to the growth and development of piglets. ALB is the most important protein in plasma, which can maintain the body’s nutrition and osmotic pressure, and has a significant role in promoting the transport of nutrients, and ALB can play a protective role in immunoglobulin, thereby exerting its immune effect (51). Treatment with different calcium sources and acidulants did not affect albumin in the current study. Hypercholesterolemia occurs when there is an elevated level of TC in the bloodstream. This can result from high levels of LDL as compared to HDL (52). Replacing calcium carbonate with calcium citrate in this study could reduce LDL in piglet blood, it indicated that calcium citrate supplementation is superior to calcium carbonate in piglet diets. In this study, the addition of various calcium sources and acidulants did not significantly improve the interleukin and tumor necrosis factor of weaned piglets, and therefore could not simultaneously modulate the immune system of piglets.

The microbiome consists of trillions of microbial cells with high inter- and intra-species variability, which makes it difficult to define a healthy gut microbiome in terms of species in the gut (53, 54). However, microbial functional genes and metabolites may have lower variability (54, 55). To study the complex relationship between the host and microbes, it is crucial to better understand the crosstalk between host and gut microbes. This can be achieved by measuring the molecules that contribute to this interaction, especially the metabolites formed by the microbiota that are available for uptake by the host. Gut microbes play a key role in animal health, including digesting food, metabolism, regulating immunity, and defending against invading pathogens (56–58). The composition of different microbial communities in the digestive tract of animals is different, and the diversity and density of microbial communities increase gradually from the stomach to the hindgut (59). We explored the effects of different calcium sources and acidulant use on gut microbial diversity in weaned piglets, using Chao1 to represent bacterial richness and the Shannon index to reflect bacterial diversity. In this study, it can be concluded that different calcium sources had no effect on the colonic microbial abundance and diversity of piglets, and both the addition of calcium citrate and organic trace elements increased the fecal microbial abundance and diversity of piglets compared with calcium carbonate diets, indicating that calcium citrate and organic trace elements are better for gut health than calcium carbonate.

The microorganisms in intestines of pig are mainly anaerobic bacteria and facultative anaerobic bacteria, of which Firmicutes and Bacteroidetes account for more than 90% and play an important role in maintaining body health and improving body immunity, nutrient absorption, and metabolism (60). This study reached similar conclusions in the colon of piglets. Much evidence suggests that Firmicutes and Bacteroidetes are the predominant phyla in pig fecal samples (61–64), and this study supports this conclusion. Diet represents one of the major factors contributing to intestinal microbial colonization (65). Similarly, a gradual taxonomic and functional rearrangement of the bacterial community in feces after feeding four different diets varying in protein source, calcium, and phosphorus concentration has been recorded (66), which indicates the importance of diet on microbial population modulation. The largest and most dynamic change in microbiome transition, however, is during the weaning period (67). Bacteria in Firmicutes are capable of producing short-chain fatty acids (SCFA) from dietary compounds (68). Li et al. reported that the intestinal community of rats had similar cecal contents and feces (69), and we found that this phenomenon was also present in the colon and feces of piglets. Previous studies have shown that some *Clostridium* species are causative agents of intestinal diseases (70), which can alter the intestinal barrier in animals (71). Our study confirmed that the addition of multiple calcium to piglet diets reduced *Clostridium_sensu_stricto_1* abundance. A study reported that 2 days post-weaning of piglets, intestinal *Lactobacillus* decreased sharply, while the number of coliforms increased (72). Bacteria in the genus *Lactobacillus* (phylum Firmicutes) are beneficial to the intestine, producing bacteriocins, organic acids, and hydrogen peroxide (73); no changes in *Lactobacillus* were found among the five treatments in this study. Replacing calcium carbonate with calcium citrate, *unclassified_f_Lachnospiraceae* abundance increased and was not affected by acidifier concentration. This suggests that the calcium citrate diet affects the gut microbial composition of weaned piglets. Likewise, multiple calcium can also alter the microbes in piglet manure compared to calcium citrate. In addition, calcium citrate and multiple calcium, instead of calcium carbonate, increased the abundance of *Phascolarctobacterium*. Different dietary components have different microbiota, which may be an explanation, as it is known that the composition of gut microbiota is related to diet type (74). High-throughput sequencing technology has many advantages. It can not only accurately analyze the structure and diversity of gut microbiota but also further predict the gene function and metabolic pathways of gut microbes. PICRUSt was used to predict the functional composition of a metagenome using marker gene data and a database of reference genomes obtained from 16S rRNA sequencing. The results of gut microbiota showed that the microbes were mainly involved in carbohydrate metabolism, amino acid metabolism, energy metabolism, and metabolism of cofactors and vitamins. Lamendella et al. showed that Firmicutes and Bacteroidetes in the intestinal microorganisms of pigs are related to carbohydrate metabolism in the body (75). According to the results of intestinal microbial function prediction, we anticipated that different calcium sources would not widely alter gut and fecal microbiota function.

## Conclusion

In conclusion, this study showed that different calcium supplementation in weaned piglet diets could affect the small intestinal barrier of piglets, but different calcium had no effect on the immune performance of piglets. At the same time, although the diarrhea of piglets was not affected by different calcium sources, the structure of fecal flora of piglets was significantly changed by different calcium sources. Our study provides a more comprehensive understanding of gut barrier in weaned piglets to different calcium sources and intestinal and fecal microbial responses, including gut and fecal bacterial composition and functional potential.

## Data Availability Statement

The datasets presented in this study can be found in online repositories. The names of the repository/repositories and accession number(s) can be found in the article/Supplementary Material.

## Ethics Statement

The studies were approved by the Laboratory Animal Welfare and Animal Experimental Ethical Inspection Committee at the Guangxi University (Nanning, China).

## Author Contributions

HS designed the experiment. KW and AY conducted the experiment. KW, AY, XP, FL, YiW, YC, YuW, and JZ collected and analyzed data. KW wrote the manuscript. HS revised the manuscript. All authors contributed to the article and approved the submitted version.

## Conflict of Interest

JZ was employed by Nanning Zeweier Feed Co., Ltd. The remaining authors declare that the research was conducted in the absence of any commercial or financial relationships that could be construed as a potential conflict of interest.

## Publisher’s Note

All claims expressed in this article are solely those of the authors and do not necessarily represent those of their affiliated organizations, or those of the publisher, the editors and the reviewers. Any product that may be evaluated in this article, or claim that may be made by its manufacturer, is not guaranteed or endorsed by the publisher.

## Footnotes

1
http://www.ncbi.nlm.nih.gov/bioproject/815982
