# Supplementary material for: Linkages of Various Calcium Sources on Immune Performance, Diarrhea Rate, Intestinal Barrier, and Post-gut Microbial Structure and Function in Piglets
Source: Front Nutr. 2022 Jun 17;9:921773. doi: 10.3389/fnut.2022.921773 (PMC9248811; doi:10.3389/fnut.2022.921773)
Supplement: Supplementary file 1 [file Table_1.DOC]

**Table S1.** Diarrhea index score of feces ^[1]^

| **Fecal appearance** | **diarrhea index** |
| --- | --- |
| hard feces | 1 |
| slightly soft feces | 2 |
| partially formed feces | 3 |
| loose, semi-liquid feces | 4 |
| watery, mucous-like feces | 5 |

[1] Li, S., Zheng, J., Deng, K., Chen, L., Zhao, X. L., & Jiang, X., et al. (2019). Supplementation with organic acids showing different effects on growth performance, gut morphology, and microbiota of weaned pigs fed with highly or less digestible diets 1 organic acids effects on health of piglets.
